# Role of XIAP gene overexpressed bone marrow mesenchymal stem cells in the treatment of cerebral injury in rats with cerebral palsy

**DOI:** 10.1186/s12935-019-0988-6

**Published:** 2019-10-23

**Authors:** Wenjing Deng, Chenghe Fan, Yanbo Fang, Yanan Zhao, Yamin Wei, Meng Li, Junfang Teng

**Affiliations:** The Neurology Intensive Care Unit, The First Affiliated of Zhengzhou University, No.1, Jianshe Road, Zhengzhou, 450052 Henan People’s Republic of China

**Keywords:** Cerebral palsy, XIAP, Bone marrow mesenchymal stem cells, Cerebral injury, Astrocytes, AchE

## Abstract

**Background:**

This study is performed to investigate the effects of adenovirus-mediated X-linked inhibitor of apoptosis protein (XIAP) overexpressed bone marrow mesenchymal stem cells (BMSCs) on brain injury in rats with cerebral palsy (CP).

**Methods:**

Rat’s BMSCs were cultured and identified. The XIAP gene of BMSCs was modified by adenovirus expression vector Ad-XIAP-GFP. The rat model of CP with ischemia and anoxia was established by ligating the left common carotid artery and anoxia for 2 h, and BMSCs were intracerebroventricularly injected to the modeled rats. The mRNA and protein expression of XIAP in brain tissue of rats in each group was detected by RT-qPCR and western blot analysis. The neurobehavioral situation, content of acetylcholine (Ach), activity of acetylcholinesterase (AchE), brain pathological injury, apoptosis of brain nerve cells and the activation of astrocytes in CP rats were determined via a series of assays.

**Results:**

Rats with CP exhibited obvious abnormalities, increased Ach content, decreased AchE activity, obvious pathological damage, increased brain nerve cell apoptosis, as well as elevated activation of astrocyte. XIAP overexpressed BMSCs improved the neurobehavioral situation, decreased Ach content and increased AchE activity, attenuated brain pathological injury, inhibited apoptosis of brain nerve cells and the activation of astrocytes in CP rats.

**Conclusion:**

Our study demonstrates that XIAP overexpressed BMSCs can inhibit the apoptosis of brain nerve cells and the activation of astrocytes, increase AchE activity, and inhibit Ach content, so as to lower the CP caused by cerebral ischemia and hypoxia in rats.

## Background

Cerebral palsy (CP) is defined as a group of permanent movement and posture disorders that results in restricted activity due to non-progressive disturbances that occur early in brain development [1, 2]. Although it is recognized that various physiological and cognitive symptoms are associated with the disease, chronic pain has been a subject of clinical and empirical concern in recent years [3, 4]. The obstruction in diagnosing the syndrome stems from its different classifications, for the reason that CP can be defined by anatomical brain damage in the cerebral cortex, extrapyramidal system, pyramidal tract, or cerebellum [5]. Routine therapies for CP include orthomorphia, neurotomy, physical therapy, language training, motor function training, and intramuscular injections of botulinum toxin A [6]. Stem cell transplantation is suggested to be a new and promising treatment for CP [7]. Nevertheless, this procedure is still at the preliminary stage of investigation and no clinical trial results have been published so far [8, 9].

Several types of stem cells are reported to be candidates for treating CP, including olfactory ensheathing cells, human embryonic neural stem cells, umbilical mesenchymal stem cells (MSCs), as well as bone marrow mesenchymal stem cells (BMSCs) [10, 11]. Autologous MSCs may be a good source of cells, which have been introduced for the treatment of CP due to their roles in tissue repair together with the regulation of immunological processes [12]. In addition, accumulating studies have indicated that human BMSCs show neural phenotypes, which is capable of differentiating into neural stem cell (NSC)-like cells in vitro [13–15]. Based on which, we hypothesized that BMSCs could be used as a novel treatment for the patients with CP. X-linked inhibitor of apoptosis protein (XIAP) is the most effective inhibitor of natural apoptosis through inhibiting the initiator (e.g. caspase-9) and effector (e.g. caspase-3 and -7) caspases along with inhibiting caspase-independent cell death [16, 17]. It is reported that the regulation of XIAP may be a promising neuroprotective strategy for treating acute and chronic neurodegenerative disorders [18]. Furthermore, a study has shown that combining XIAP with MSC-delivered soluble tumor necrosis factor-related apoptosis inducing ligand (sTRAIL) contributed to the regression of primary tumors without lacking of metastases [19]. Saito et al. have demonstrated that XIAP plays an significant role in the control of the death of apoptotic neuronal cells after transient focal cerebral ischemia [20]. Furthermore, XIAP is of great importance in the apoptosis resistance of HL-60 cells when co-cultured with BMSCs through direct cell contact, and the inhibition of XIAP provides a new strategy for treating acute myeloid leukemia [21]. Therefore, we conducted this study to investigate the effects of adenovirus-mediated XIAP modification of BMSCs on brain injury in rats with CP.

## Materials and methods

### Ethics statement

The study was approved by the animal ethics committee in The First Affiliated of Zhengzhou University (approval number: 2019-KY-220). All animal work was conducted to relieve their pain according to relevant national and international guidelines.

### Experimental animals

A total of 73 Sprague–Dawley (SD) rats in clear grade (regardless of gender, aging 7 days and weighting 20 ± 5 g) were purchased from Beijing Vital River Laboratory Animal Technology Co., Ltd. (Beijing, China). The rats had free access to eating and drinking in a room with 12 h day/night cycle, temperature of (23–25) °C, humidity of 45–60% as well as regular disinfection and ventilation.

### Culture and identification of BMSCs in rats

One rat was euthanized by cervical dislocation, and immersed in 75% ethanol for 5 min. The tibia and femur were separated in aseptic condition, and the muscle and fibrous tissues were removed. The marrow cavity was washed several times in DMEM/DF12 medium (Gibco, Grand Island, NY, USA) with a 1 mL injector, until the marrow cavity became white and bright. The cells were suspended and inoculated into a culture flask with the concentration of 1 × 10^6^ cells/mL and incubated in an incubator at 37 °C with 5% CO_2_ for 3 days. When the adherent cells reached 80–90% confluence, the culture medium was removed. The cells were detached with 0.25% trypsin at room temperature and sub-cultured in the ratio of 1:2. Afterwards, the culture process was repeated every 2 to 3 days, when the cell confluence reached 80–90%, the sub-culture process was repeated.

The cultured and amplified rat’s BMSCs at passage 3 were detached with 2.5 mL trypsin, then washed with PBS containing 10 g/L bovine serum albumin (BSA). The cells were suspended for preparing the single cell suspension at a density of 2 × 10^5^ cells/mL. The cells were added with rabbit anti-mouse CD29, CD44, CD34 and CD4 (each 5 μL) and homotypic control flow antibody and incubated at room temperature in dark for 15–20 min. The cells were detected by a flow cytometer (Becton, Dickinson and Company, Franklin Lake, New Jersey, USA).

When the cells in the culture dish reached 80–90% confluence, the cells were seeded to the six-well plate, and cultured in the incubator. After reaching 70% cell confluence, the original culture medium was removed and then cultured in osteogenic differentiation medium (2 mL/well, Guangzhou Saiye Biotechnology Co., Ltd., Guangzhou, Guangdong, China). The liquid was changed once every 3 days, five times in total. After abandoning the original culture medium, the cells were fixed with 10% neutral formaldehyde solution, stained with 1 mL alizarin red S (Cyagen Biosciences, Santa Clara, CA, USA) for 5 min. The staining was observed under the microscope.

When the cells in the culture dish reached 80–90% confluence, the cells were inoculated to the six-well plate, and cultured in the incubator. After reaching 100% cell confluence, the original culture medium was removed and then cultured in adipose induced differentiation medium (2 mL/well, Guangzhou Saiye Biotechnology Co., Ltd., Guangzhou, Guangdong, China). After 3 days, the medium was replaced by growth medium and then changed into adipose induced differentiation medium after 24 h. The above two medium were used alternately, with a total of three to five times exchange. The cells were fully cultured for about a week, in which the medium solution was changed every 3 days. After abandoning the original culture medium, the cells were fixed with 10% neutral formaldehyde solution, stained with 1 mL Oil red o solution (Cyagen Biosciences, Santa Clara, CA, USA) for 30 min. The staining was observed under the microscope.

### BMSCs transfected with adenovirus expression vector Ad-XIAP-green fluorescent protein (GFP)

BMSCs was inoculated in a 24-well plate at the density of 1.5 × 10^5^ cells/well and cultured for 24 h. The Ad-XIAP-GFP virus solution with the multiplicity of infection (MOI) of 100, 200 and 400 were transferred into the BMSCs. After 12 h, the solution was changed and the complete culture medium was added for further culture. The expression of GFP in BMSCs transfected with Ad-XIAP/GFP was observed by a fluorescence microscope to determine the MOI. In order to verify the transfection efficiency, the cells which were infected with the MOI of Ad-XIAP/GFP and Ad-GFP on the 3rd day and the untreated cells were detected by reverse transcription quantitative polymerase chain reaction (RT-qPCR) and western blot analysis to detect the expression of XIAP mRNA and protein in cells. The BMSCs that successfully infected the recombinant adenoviral vector carrying the XIAP gene (Ad-GFP-XIAP) were named XIAP-BMSCs. BMSCs infected with empty vector virus (Ad-GFP) were named NC-BMSCs, and uninfected BMSCs were named as blank. Both Ad-GFP-XIAP and Ad-GFP expression vectors were constructed and identified by Shanghai Sangon Bioengineering Co., Ltd. (Shanghai, China).

### Establishment of a rat model of CP

The rats were numbered according to their body weight and 12 rats were randomly selected as a sham group. The rat model of CP was established by ligating the left common carotid artery and anoxia for 2 h [22] in the remaining 60 rats. The anesthetized rats were intraperitoneally injected with 1% pentobarbital sodium for anesthesia. The experimental rats were in supine on the surgical plate, fixed their limbs and heads, and disinfected with iodophor before the neck. The left common carotid artery was found in the triangle area at the junction of the inner side of the sternocleidomastoid and the anterior part of the neck, and the incision was sutured with a No. 0 wire. The experimental rats were immediately placed in an anoxic chamber at 37 °C. The mixture of 8% oxygen and 92% nitrogen was continuously filled with the flow rate of 1 L/min for 2 h. The whole process of anoxia was continuously monitored by an oxygen meter. In the sham group, the common carotid artery was isolated, and no ligation was performed without hypoxia treatment.

### Experimental grouping and treatment

The successfully-modeled rats were divided into 5 groups, 12 rats in each group: CP group (establish a rat model of ischemic and anoxic CP without other treatment), CP + PBS group (on the 3rd day after modeling, stereotactic injection of 2 μL PBS into the left lateral ventricle of CP rats), CP + BMSCs group (on the 3rd day after modeling, stereotactic injection of 2 μL BMSCs into the left lateral ventricle of CP rats), CP + NC-BMSCs group (on the 3rd day after modeling, stereotactic injection of 2 μL NC-BMSCs into the left lateral ventricle of CP rats), and CP + XIAP-BMSCs group (on the 3rd day after modeling, stereotactic injection of 2 μL XIAP-BMSCs into the left lateral ventricle of CP rats). The rats in the CP + PBS group, CP + BMSCs group, CP + NC-BMSCs group and CP + XIAP-BMSCs group were fixed on the stereotactic locator on the 3rd day after modeling. The scalp sagittal incision was made under aseptic operation, and 2 μL PBS, BMSCs, NC-BMSCs and XIAP-BMSCs (about 5 × 10^5^ BMSCs) were injected into the left ventricle via a microinjector.

### Evaluation of nerve function injury

Neurobehavioral assessment of rats was conducted at 3 weeks after modeling.

Morris water maze test: The rats facing the water wall were placed in water several times from 4 water entry points to record the time when they found the hidden platform under the water surface, namely the escape latency.

Suspension test: The rat forelegs grabbed the 0.5 cm glass rod and observed the time of falling: < 10 s was 1 point, 10–30 s was 2 points, 31–119 s was 3 points, 2–5 min was 4 points, > 5 min was 5 points.

Slope test: The rats were placed on a 45° slope to observe the turning time.

Open field test: The covered box with length and width of 36 cm was divided into 9 equal size lattices at the bottom of the box, and then to record the activity of rats. More than 1/2 of the body part of the rat entered the adjacent grid was 1 point, and the hind limb of the rat stood was 1 point. The sum of the two points was the total score.

### Hematoxylin–eosin (HE) staining

After the neurological deficit score was finished, 8 rats were taken from each group, and the brain was removed from the neck, and then the brain tissue was quickly separated on the ice. The brain tissue of 4 rats was placed in a prelabeled cryopreservation tube and rapidly put into liquid nitrogen, and then transferred to − 80 °C for the detection of RT-qPCR and western blot analysis. In addition, the brain tissues of 4 rats in each group were fixed in 4% paraformaldehyde for 24 h, dehydrated, embedded, and sliced with the thickness of 4 μm, which were used for HE staining, terminal deoxynucleotidyl transferase-mediated dUTP nick end-labeling (TUNEL) staining and immunohistochemical staining.

According to the isometric random method, each group of rats was randomly selected with five slices of brain tissue. The slices were dewaxed with xylene for 20 min, and hydrated with 100%, 95%, 80% and 70% for 5 min, respectively, stained with hematoxylin (Beijing Solarbio Science & Technology Co., Ltd., Beijing, China) for 4 min, differentiated with hydrochloric acid alcohol and washed with running water for 5 min. Next, the slices were stained with eosin (Beijing Solarbio Science & Technology Co., Ltd., Beijing, China) for 3 min, followed by gradient alcohol dehydration, xylene clearance, and sealing with neutral gum. Tissue staining was observed under an optical microscope (DSX100, Olympus, Tokyo, Japan).

### TUNEL staining

According to the isometric random method, 5 slices of brain tissue were randomly selected from each rat in each group and immersed in 200 mL 0.1 mol/L sodium citrate buffer solution for 5 min under microwave heating, and then poured into 80 mL distilled water immediately. Next, the tissues were added with TUNEL solution ((Boster Biological Technology, Ltd., Wuhan, China) and reacted at 37 °C for 1 h. After that, the tissues were supplemented with converter-POD and reacted at 37 °C for 1 h. Lastly, the tissues were counterstained with hematoxylin, followed by gradient alcohol dehydration, xylene clearance, and sealing with neutral gum. The positive cells in the left brain region were counted when the brown granules were stained. Apoptotic index (AI) = the number of positive cells/the total number of cells × 100%.

### Immunohistochemical staining

Five slices of brain tissue were randomly selected from each rat in each group and rinsed with PBS for three times, each time for 10 min, fixed with 4% 1-phosphofructaldolase (PFA) for 10 min, and rinsed with PBS for three times, each time for 5 min. After sealing with 5% goat serum, the tissues were added with primary antibody, glial fibrillary acidic protein (GFAP) (1:200, Abcam, Cambridge, MA, USA) and incubated at 37 °C for 2 h. Next, the tissues were rinsed with PBS for three times, each time for 10 min, supplemented with S-P compound and incubated at 37 °C for 30 min. Afterwards, the tissues were rinsed with PBS for three times, each time for 10 min, supplemented with 0.05% diaminobenzidine (DAB) solution, followed by alcohol dehydration, xylene clearance, and sealing with neutral gum. Under the same exposure condition, the images were analyzed by software Image-Pro Plus 6.0 (Media Cybernetics, Rockville, Maryland, USA), and 5 fields of vision were randomly selected to count the average number of positive cells of GFAP.

### Transmission electron microscope observation

The remaining 4 rats in each group were anesthetized by intraperitoneal injection of 1% pentobarbital sodium after neurobehavioral evaluation. The heart was exposed by opening the chest, and the left ventricle was inserted into the ascending aorta by cutting the left ventricle into the ascending aorta and fixed with hemostatic forceps. The abdominal aorta was clamped with hemostatic forceps. The right atrium was cut open and 50 mL normal saline containing 12.5 U/L heparin sodium was quickly infused. After the blood was washed out, 2% glutaraldehyde was perfused with 100–150 mL glutaraldehyde. About 1 mm^3^ ischemic brain tissue mass was fixed with 4% glutaraldehyde for 4 h and 1% osmium acid for 2 h, followed by gradient ethanol dehydration, epoxy resin 618 embedding as well as uranium acetate and lead citrate electron staining. The brain cells were observed under a JEM-1400Plus electron microscope (JEOL, Tokyo, Japan).

### RT-qPCR

The Trizol one-step method (Invitrogen, Carlsbad, CA, USA) was implemented to extract the total RNA in brain tissues and BMSCs. The complementary DNA (cDNA) was obtained by avian myeloblastosis virus (AMV) reverse transcriptase after obtaining l μg RNA. SYBR Green was used for qPCR, and glyceraldehyde phosphate dehydrogenase (GAPDH) was selected as an internal control. PCR primer was designed and synthesized by Invitrogen (Carlsbad, CA, USA) (Table 1). RT-qPCR instrument (ABI 7500, ABI, Foster City, CA, USA) was used for detection. The 2^−ΔΔCt^ method was used to analyze the ratio relation of target gene expression between the experimental group and the control group. The experiment was repeated in triplicate.

**Table 1:** Primer sequence

Gene	Sequence
XIAP	F: 5′-CCCTTGGGAACAGCATGCTA-3′
	R: 5′-AATCCAGCACCACAGTAGGC-3′
GFAP	F: 5′-AGGCCTAGGCATCTGGAAGA-3′
	R: 5′-ATCCTTCTGAGGCCCTCCAT-3′
GAPDH	F: 5′-CAGCCGCATCTTCTTGTGC-3′
	R: 5′-GGTAACCAGGCGTCCGATA-3′

*F* forward, *R* reverse, *XIAP* X-linked inhibitor of apoptosis protein, *GFAP* glial fibrillary acidic protein, *GAPDH* glyceraldehyde phosphate dehydrogenase

### Western blot analysis

The proteins from brain tissues and BMSCs were extracted and the protein concentrations were determined referring to the instructions of the bicinchoninic acid assay (Boster Biological Technology, Ltd., Wuhan, China). After the proteins were separated by 10% sodium dodecyl sulfate polyacrylamide gel electrophoresis (Boster Biological Technology, Ltd., Wuhan, China), they were transferred to a nitrocellulose membrane using the wet transfer method. Subsequently, the protein samples were transferred to polyvinylidene fluoride membrane and blocked with 5% BSA for 1 h. Afterwards, the membranes were added with the primary antibodies to XIAP (1: 1000, ab229050), cleaved caspase-3 (1:500, ab49822), Bax (1:1000, ab53154), Bcl-2 (1:1000, ab196495) and β-actin (1:1000, ab8227). All these antibodies were purchased from (Abcam, Cambridge, MA, USA) and incubated at 4 °C overnight. The membranes were then rinsed with TBST for 3 times, each time for 10 min. The corresponding IgG secondary antibody labeled by horseradish peroxidase (1: 2000, ab6721, Abcam, Cambridge, MA, USA) were incubated for 2 h at room temperature so as to wash the membranes for 3 times, each time for 10 min. After DAB coloration, a gel imager was used for photographing (Gel Doc XR, Bio-Rad, Hercules, CA, USA). The gray value analysis of target band was analyzed by Image J software (National Institutes of Health, Bethesda, Maryland, USA).

### Enzyme-linked immunosorbent assay (ELISA)

The brain tissue was thawed at room temperature and weighed it accurately. The content of acetylcholine (Ach) and the activity of acetylcholinesterase (AchE) in the homogenate of rat brain tissue were determined by ELISA kit (Nanjing Jiancheng Institute of Bioengineering, Nanjing, Jiangsu, China) after homogenization in tissue lysate.

### Statistical analysis

All statistical analyses were performed using the SPSS 21.0 software (IBM SPSS, Inc., Chicago, IL, USA). The data were normally distributed by Kolmogorov–Smirnov test. The measurement data were expressed as mean ± standard deviation. The t test was used for the comparison between the two groups, and one-way analysis of variance (ANOVA) was used for the comparison among multiple groups. Fisher’s least significant difference t test (LSD-t) was used for pairwise comparisons after ANOVA analysis. *P* values ≤ 0.05 were considered statistically significant.

## Results

### Morphological observation and identification of BMSCs

**Fig. 1:**
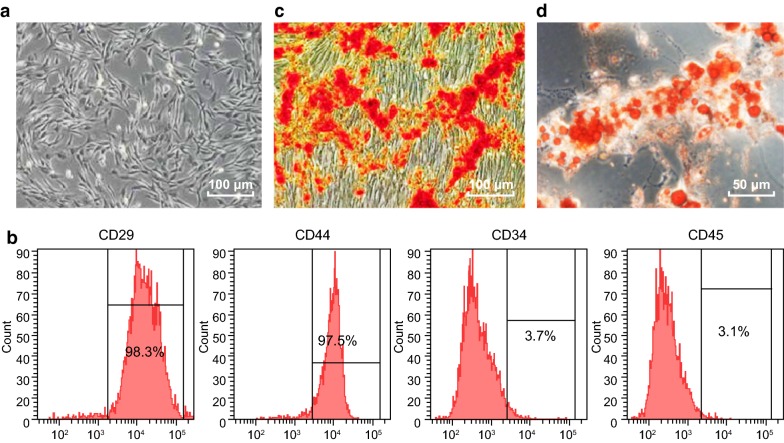
Morphological observation and identification of rat BMSCs. **a** The morphology of cells at passage 3 by an inverted microscope (×100). **b** Identification of cell surface markers by flow cytometry. **c** Identification of osteogenic differentiation ability of cells by alizarin red staining (×100). **d** Identification of adipogenic differentiation ability of cells by oil red O staining (×200)

During the initial isolation of rat BMSCs, most of the cells were round in shape, a small number of cells showed irregular shape, and a few blood cells were mixed with them. The cells were suspended in the cell culture medium and varied in size. After 8 h of culture, some cells were observed to be adhered to the wall, and then to show spindles and strips after 72 h. When the cells were cultured, the cells gradually converged, and the size of the cells increased gradually. After the growth and purification of adhered cells, the proliferation rate of cell culture increased, and the morphology and size of the cells gradually unified, showing a fiber strip (Fig. 1a).

Specific surface markers of isolated cells were identified by flow cytometry. The results showed that surface markers of MSCs, CD29 (98.3%) and CD44 (97.5%) [23], were highly expressed while surface markers of hematopoietic stem cells, CD34 (3.7%) and CD45 (3.1%) [23], were lowly expressed in cultured rat BMSCs, which were identified as the immunophenotypic characteristics of BMSCs (Fig. 1b).

After differentiation of cultured osteoblasts, a large number of calcium nodules could be seen under an alizarin red staining microscope (Fig. 1c), showing that the cells have good osteogenic differentiation function. After the intervention of the fat-inducing agent, the red-stained particles could be seen in the cytoplasm of the cells through the oil-red O staining, and a large amount of lipid droplets were visible (Fig. 1d), suggesting the existence of adipose differentiation in cells. These results suggest that rat BMSCs were successfully cultured.

### Infection efficiency of adenovirus expression vector Ad-XIAP-GFP

**Fig. 2:**
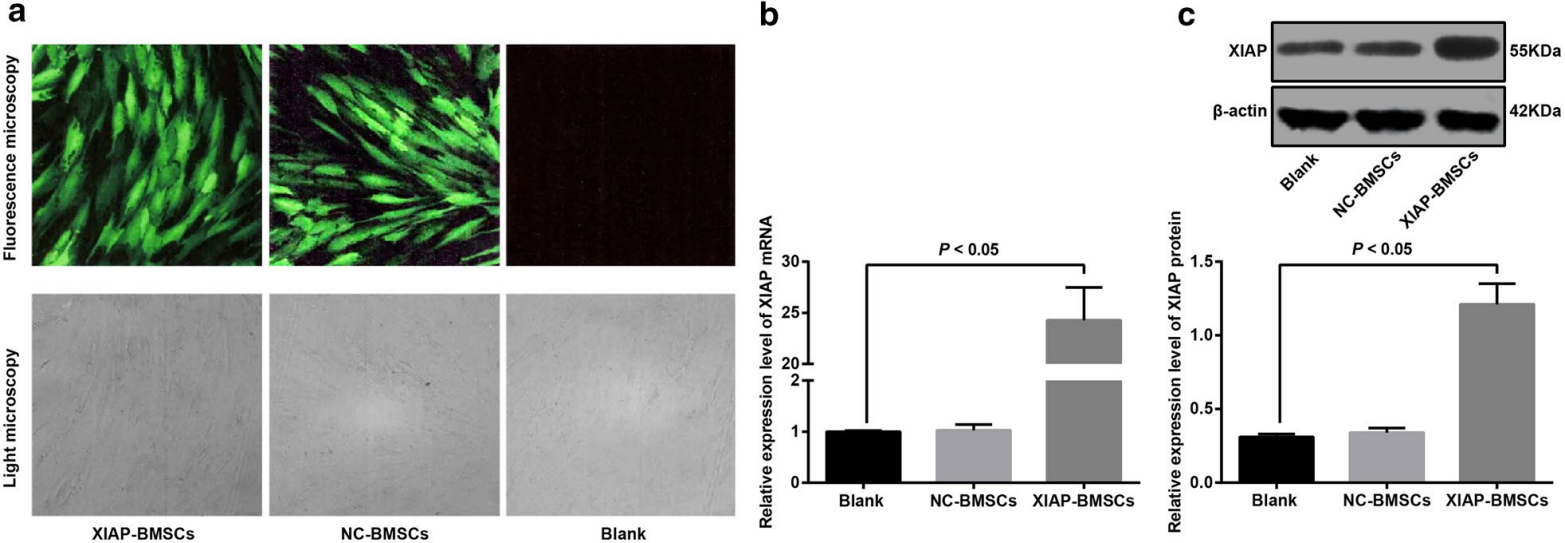
Identification of adenovirus expression vector Ad-XIAP-GFP and expression of XIAP in cells of each group. **a** Observation of infection effect in each group under the fluorescence microscope and light microscope (The BMSCs that successfully infected the recombinant adenoviral vector carrying the XIAP gene (Ad-GFP-XIAP) were named XIAP-BMSCs. BMSCs infected with empty vector virus (Ad-GFP) were named NC-BMSCs, and uninfected BMSCs were named as blank). **b** Detection of mRNA expression of XIAP in cells by RT-qPCR. **c** Detection of XIAP protein expression by western blot analysis. The experiment was repeated three times. Data among multiple groups was analyzed by one-way ANOVA, and LSD-t was conducted in pairwise comparison after ANOVA

Under an inverted phase contrast microscope, the MSCs infected by the virus was spindle-shaped, with a large nucleus, oval or round, similar to that of uninfected BMSCs. Under a fluorescence microscope, the BMSCs infected by the virus showed green fluorescence in both nucleus and cytoplasm, and the fluorescence intensity was uneven. A small amount of green fluorescence appeared 24 ho after transfection, and the fluorescence increased obviously at 72 h. The infection efficiency was over 90%, and the best transfection efficiency was MOI = 200. The infection efficiency of XIAP-BMSCs was similar to that of NC-BMSCs (92.04% vs. 90.47%) (Fig. 2a).

In order to investigate the expression of XIAP after Ad-XIAP-GFP infection with BMSCs, the cells infected with Ad-XIAP-GFP and Ad-GFP on the 3rd day and the untreated cells were selected, and the expression of XIAP mRNA and protein was detected by RT-qPCR and western blot analysis. The results showed that XIAP mRNA and protein expression in NC-BMSCs (BMSCs infected with empty vector virus [Ad-GFP]) had no significant change compared with the blank BMSCs (uninfected BMSCs) (*P* > 0.05). The mRNA and protein expression of XIAP in XIAP-BMSCs (BMSCs that successfully infected the recombinant adenoviral vector carrying the XIAP gene [Ad-GFP-XIAP]) increased significantly compared with the blank BMSCs (uninfected BMSCs) (*P* < 0.05), indicating that XIAP modified BMSCs was successfully constructed (Fig. 2b, c).

### XIAP modified BMSCs up-regulated the expression of XIAP in the brain tissues of CP rats

**Fig. 3:**
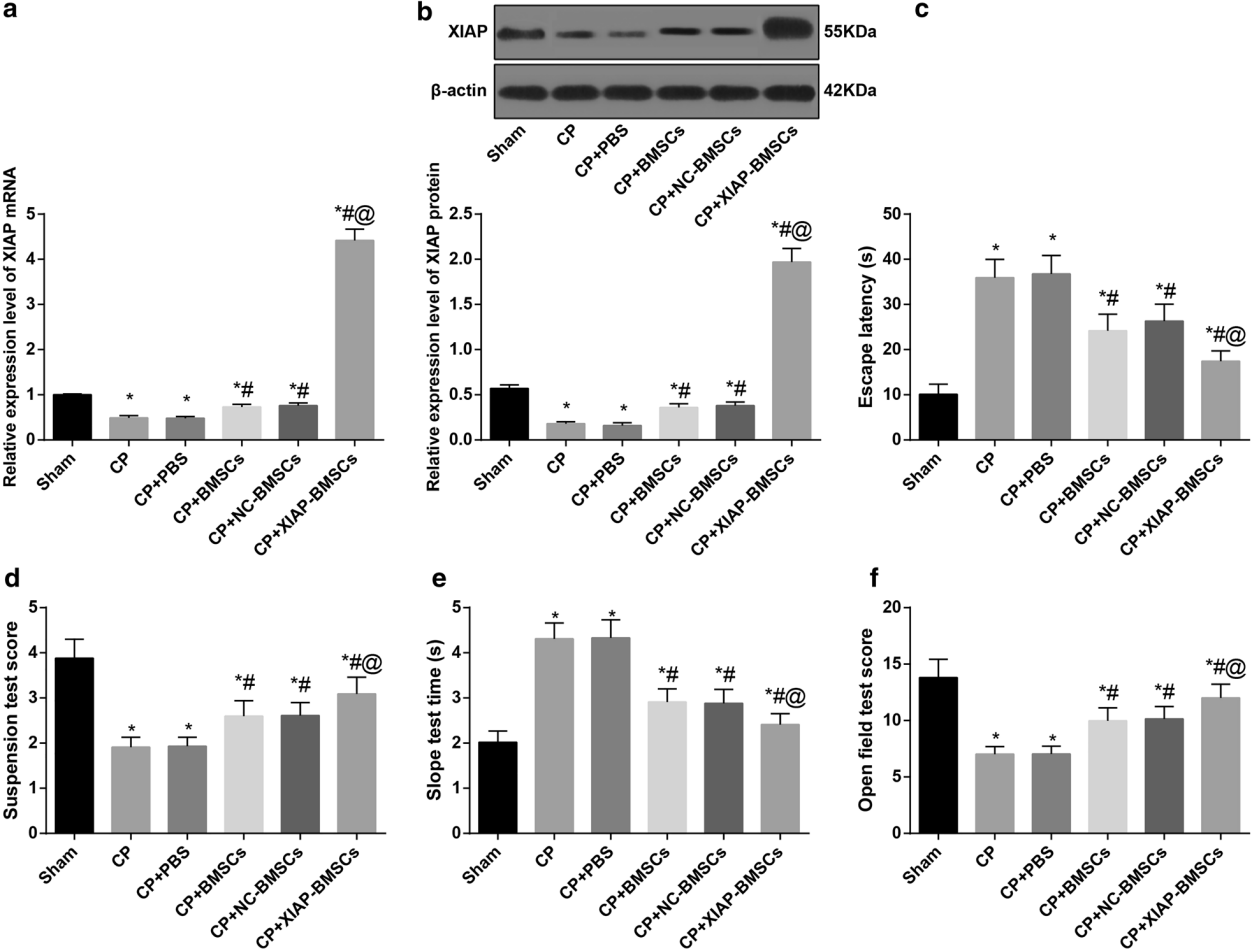
BMSCs modified by XIAP improves the neurobehavioral situation in CP rats. **a** Detection of mRNA expression of XIAP in brain tissues of rats by RT-qPCR, N = 4. **b** Detection of XIAP protein expression in brain tissues of rats by western blot analysis, N = 4. **c** Detection of escape latency in rats by Morris water maze test, N = 12. **d**–**f** The neurobehavioral situation analyzed by suspension test, slope test and open field test, N = 12. *P < 0.05 vs the sham group; ^#^*P* < 0.05 vs the CP group; ^@^*P* < 0.05 vs the CP + BMSCs group. Data among multiple groups was analyzed by one-way ANOVA, and LSD-t was conducted in pairwise comparison after ANOVA

RT-qPCR and Western blot analysis were used to detect the expression of XIAP in brain tissues of rats in each group. The expression of XIAP mRNA and protein in brain tissues of rats in the CP group was significantly lower than that in the sham group (*P* < 0.05), suggesting that the expression of XIAP was related to the occurrence of CP. The expression of XIAP mRNA and protein in brain tissues of rats in the CP + XIAP-BMSCs group was significantly higher than that in other CP groups (all *P* < 0.05). The results showed that XIAP modified BMSCs successfully up-regulated the expression of XIAP in the brain tissues of CP rats (Fig. 3a, b).

### BMSCs modified by XIAP improves the neurobehavioral situation in CP rats

The experimental results of Morris water maze test indicated that compared with the sham group, the escape latency of rats in the CP group and the CP + PBS group was significantly prolonged (both *P* < 0.05). Relative to the CP group and the CP + PBS group, the escape latency of rats was significantly shortened in the CP + BMSCs group and the CP + NC-BMSCs group, and that in the CP + XIAP-BMSCs group was further shortened compared with the CP + BMSCs group and the CP + NC-BMSCs group (all *P* < 0.05) (Fig. 3c).

The results of suspension test suggested that the suspension test score in rats of the CP groups was lower than that in the sham group; among the CP rats, the CP + XIAP-BMSCs group had the lowest score of suspension test, followed by the CP + BMSCs group and the CP + NC-BMSCs group, which were significantly lower than those in the CP group and CP + PBS group (all *P *< 0.05) (Fig. 3d).

The results of slope test indicated that after the establishment of CP model, the slope test time of rats was obviously prolonged (*P* < 0.05). After transplantation of BMSCs in CP rats, the slope test time of rats was significantly shortened, while that in rats after transplantation of BMSCs expressing XIAP was further shortened (all *P* < 0.05) (Fig. 3e).

The results of open field test showed that the open field score in the CP group and CP + PBS group was lower than that in the sham group. After transplantation of BMSCs, the scores of open field test were significantly decreased in rats, and those in the CP + XIAP-BMSCs group were significantly lower than those in the CP + BMSCs group and the CP + NC-BMSCs group (all *P* < 0.05) (Fig. 3f).

### BMSCs modified by XIAP decreases content of Ach and increases activity of AchE in CP rats

**Fig. 4:**
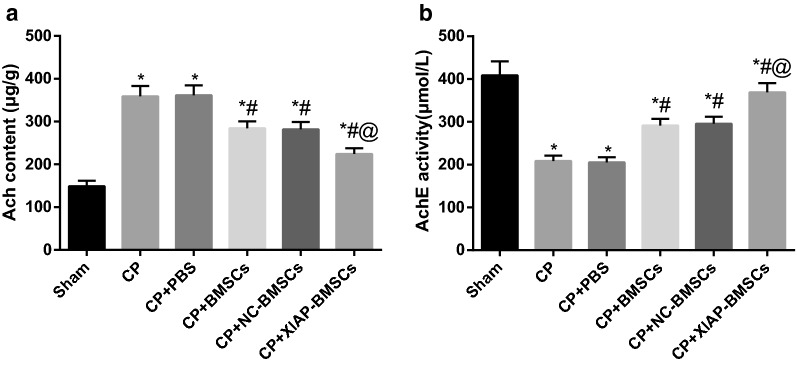
BMSCs modified by XIAP decreases content of Ach and increases activity of AchE in CP rats. **a** Detection of Ach content in brain tissues of rats by ELISA, N = 4. **b** Detection of activity of AchE in brain tissues of rats by ELISA, N = 4. *P < 0.05 vs the sham group; ^#^*P* < 0.05 vs the CP group; ^@^*P* < 0.05 vs the CP + BMSCs group. Data among multiple groups was analyzed by one-way ANOVA, and LSD-t was conducted in pairwise comparison after ANOVA

The content of Ach and the activity of AchE in brain tissues of rats were determined by ELISA to further evaluate the brain nerve injury in rats. Compared with the sham group, increased content of Ach and decreased activity of AchE were found in the brain tissues of rats in the CP and CP +PBS groups (both *P* < 0.05). Relative to the CP group, the content of Ach was decreased and the activity of AchE was increased in brain tissues of rats in the CP + NC-BMSCs group and the CP + XIAP-BMSCs group (all *P* < 0.05). In contrast to the CP + BMSCs group, the content of Ach was further decreased and the activity of AchE was further increased in brain tissues of rats in the CP + XIAP-BMSCs group (both *P* < 0.05) (Fig. 4).

### BMSCs modified with XIAP attenuates brain pathological injury in CP rats

**Fig. 5:**
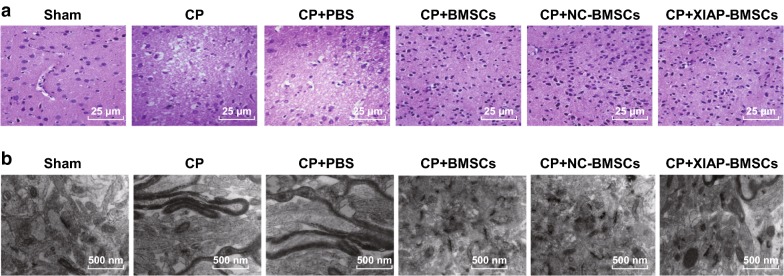
BMSCs modified with XIAP attenuates brain pathological injury in CP rats. **a** Pathological observation of brain tissue in rats by HE staining. **b** Observation of ultrastructure of rat brain by a transmission electron microscope; N = 4

The pathological morphology of brain tissues in rats was observed by HE staining. The results of which suggested that in rats of the sham group, brain tissue structure was normal, interstitial edema was not observed, cytoplasm was abundant and nucleus were clearly visible. In rats of the CP group and the CP + PBS group, the brain tissue structure was destroyed, white matter was disordered, which was presented with multiple irregular cystic cavities, cell swelling, degeneration, disordered arrangement, inflammatory cell infiltration and local round softening foci. In rats in the CP + BMSCs group and the CP + NC-BMSCs group, the degree of cell swelling was reduced, inflammatory infiltrating cells were decreased, and cystic degeneration was decreased. In rats of the CP + XIAP-BMSCs group, the pathological degree of brain tissue was further alleviated and the cells were arranged neatly (Fig. 5a).

The ultrastructure of brain tissues of rats was observed by a transmission electron microscope. In the sham group, the neuronal cells of the brain were intact, with abundant rough endoplasmic reticulum, free ribosome and mitochondria, clear mitochondrial ridge, regular arrangement, clear nuclear membrane, dominant chromatin in nucleus and obvious nucleolus. In rats of the CP group and the CP + PBS group, neuronal edema was observed, the number of organelles decreased, most of mitochondria crest and membrane fused or disappeared, some mitochondria became empty, rough endoplasmic reticulum expanded obviously, and degranulation was obvious. Free ribosome reduced, partial double layer nuclear membrane was fused and blurred. In the CP + BMSCs group and the CP + NC-BMSCs group, the neurons were slightly edema, the nuclear membrane was intact, only a small amount of shrinkage was found, the nucleus was slightly pyknotic, the chromatin was evenly distributed in the nucleus, and the cytoplasmic organelles were normal. In the CP + XIAP-BMSCs group, the edema state of neurons disappeared, the number of organelles increased significantly, the mitochondrial cristae were broken down, and the vacuolar changes were obviously improved (Fig. 5b).

### XIAP modified BMSCs inhibits apoptosis of brain nerve cells in CP rats

**Fig. 6:**
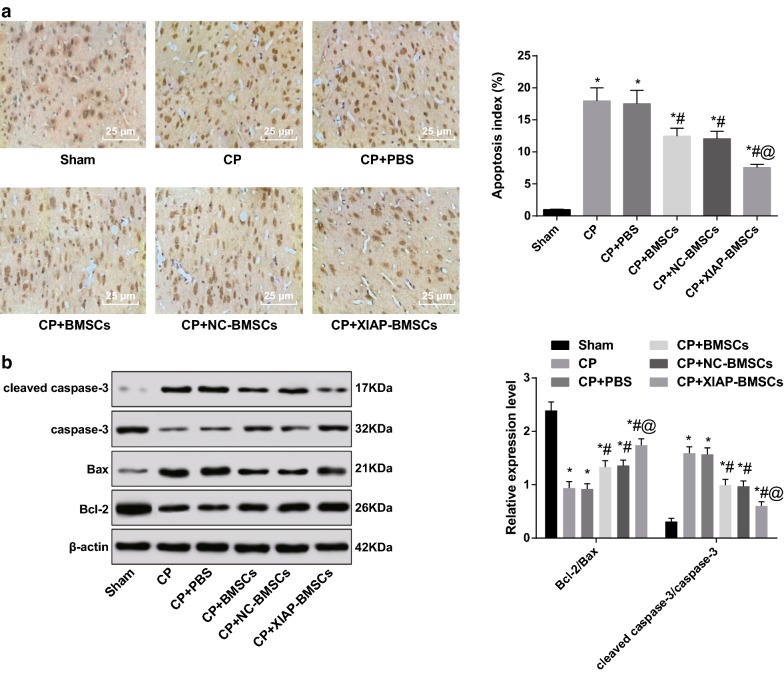
XIAP modified BMSCs inhibits apoptosis of brain nerve cells in CP rats. **a** TUNEL staining used to detect the apoptosis of brain nerve cells in rats. **b** The expression of apoptosis-related protein cleaved caspase-3, caspase-3, Bax and Bcl-2 detected by western blot analysis. N = 4. *P < 0.05 vs the sham group; ^#^*P* < 0.05 vs the CP group; ^@^*P* < 0.05 vs the CP + BMSCs group. Data among multiple groups was analyzed by one-way ANOVA, and LSD-t was conducted in pairwise comparison after ANOVA

TUNEL staining was used to detect the apoptosis of brain nerve cells in rats. The results showed that there were fewer apoptotic cells in brain tissue of rats in the sham group. The AI of the brain nerve cells in the CP and the CP + PBS groups was significantly higher than that in the sham group (all *P* < 0.05). Compared with the CP group and the CP + PBS group, the AI of brain nerve cells in the CP + BMSCs group and the CP + NC-BMSCs group was significantly decreased (all *P* < 0.05). Relative to the CP + BMSCs group and the CP + NC-BMSCs group, the AI of brain nerve cells in the CP + XIAP-BMSCs group was further decreased (all *P* < 0.05) (Fig. 6a).

Furthermore, the expression of apoptosis-related protein cleaved caspase-3, caspase-3, Bax and Bcl-2 was detected by western blot analysis. The expression of cleaved caspase-3/caspase-3 was significantly higher while the expression of Bcl-2/Bax was significantly lower in the brain tissues of rats in the CP group than that in the sham group (all *P* < 0.05). However, the expression of cleaved caspase-3/caspase-3 was significantly lower in the brain tissues of rats in the CP + BMSCs group than that in the CP group, and the expression of Bcl-2/Bax was significantly increased (all *P* < 0.05). The expression of cleaved caspase-3/caspase-3 in brain tissues of rats in the CP + XIAP-BMSCs group was significantly lower and the expression of Bcl-2/Bax was significantly higher than that in the CP + BMSCs group and the CP + NC-BMSCs group (all *P* < 0.05) (Fig. 6b).

### XIAP modified BMSCs inhibits the activation of astrocytes in CP rats

**Fig. 7:**
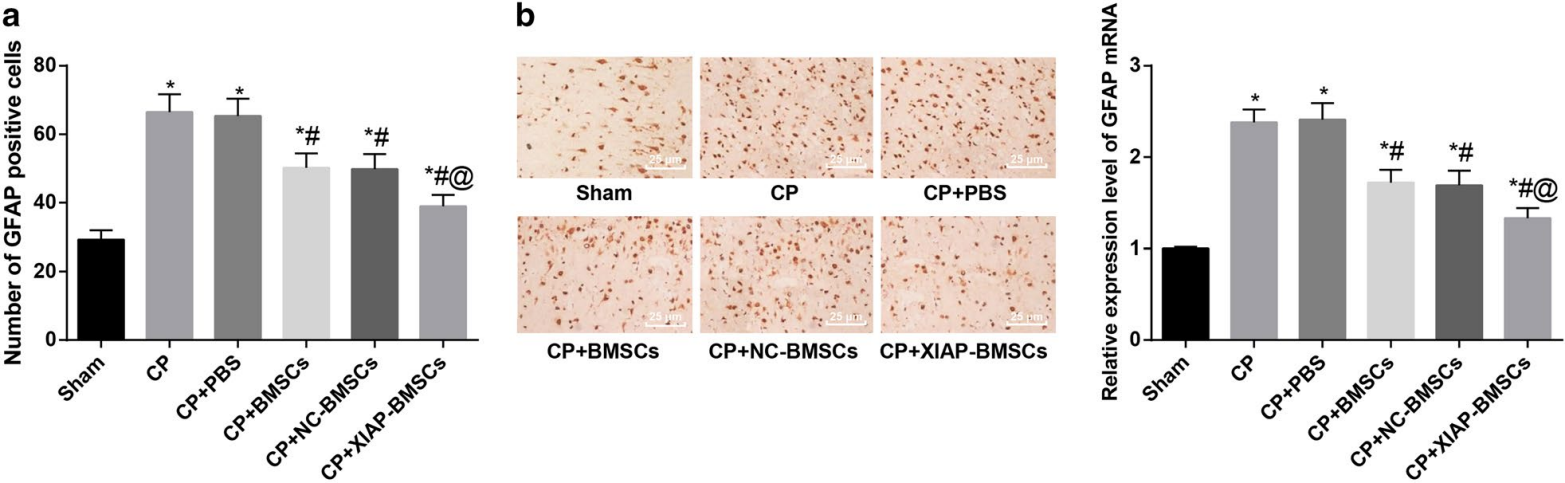
XIAP modified BMSCs inhibits the activation of astrocytes in CP rats. **a** RT-qPCR used to detect the expression of GFAP in the brain tissues of rats in each group. **b** GFAP immunohistochemistry used to detect the positive expression of GFAP in brain tissues of rats. N = 4. *P < 0.05 vs the sham group; ^#^*P* < 0.05 vs the CP group; ^@^*P* < 0.05 vs the CP + BMSCs group. Data among multiple groups was analyzed by one-way ANOVA, and LSD-t was conducted in pairwise comparison after ANOVA

RT-qPCR was used to detect the expression of GFAP, a specific marker of astrocytes, in the brain tissues of rats in each group. The expression of GFAP mRNA in brain tissues of rats in the CP group was significantly higher than that in the sham group (*P* < 0.05). There was no significant difference in the expression of GFAP mRNA between the CP group and the CP + PBS group as well as between the CP + BMSCs group and the CP + NC-BMSCs group (all *P* > 0.05). Compared with the CP group, the expression of GFAP mRNA was significantly decreased in the CP + BMSCs group and the CP + NC-BMSCs group (both *P* < 0.05), and that in the CP + XIAP-BMSCs group was further decreased compared with that in the CP + BMSCs group (*P* < 0.05) (Fig. 7a).

GFAP immunohistochemistry was used to detect the positive expression of GFAP in brain tissues of rats, so as to explain the activation of astrocytes. The results showed that the number of GFAP positive cells in brain tissues of rats in the CP and the CP + PBS groups was significantly higher than that in the sham group, while the number of GFAP positive cells in the CP + BMSCs and the CP + NC-BMSCs group was significantly lower than that in the CP group (all *P* < 0.05). The number of GFAP positive cells in brain tissues of rats in the CP + XIAP-BMSCs group was significantly lower than that in the CP + BMSCs group (*P* < 0.05) (Fig. 7b).

## Discussion

The neonatal mortality rate was decreased due to the improvements in perinatal emergency medicine, while the incidence of premature CP and hypoxic ischemic encephalopathy has increased over time [6]. Routine rehabilitation treatment for CP is unable to improve the motor function of those patients who with moderate-to-severe chronic CP [24, 25]. Previously, MSCs have been considered as an important experimental tumor therapy based on their intrinsic anticancer properties or integrated with the expression of therapeutic transgenes [19]. In this present study, we aim to investigate the effects of lentivirus-mediated XIAP modification of BMSCs on brain injury in rats with CP. Collectively, the results of this study highlights that BMSCs modified by XIAP can inhibit the apoptosis of brain nerve cells and the activation of astrocytes and increase the activity of AchE, so as to lower the CP caused by cerebral ischemia and hypoxia in rats.

Both cell type and cell modifications play a role in affecting the cells homing after intravascular delivery [26]. The application of BMSCs is particularly promising for the reason that they home to both the injured tissue or secrete factors that induce brain repair [27]. It has been demonstrated that cellular engineering, cell culture conditions as well as cell surface modifications may also regulate cells’ migratory behavior and immunomodulatory properties and further improve their therapeutic potential [28, 29]. Experiments have revealed that transplanted NSCs show strong plasticity, which is able to integrate with host cells and also become functional substituting nerve cells by establishing a stable synaptic connection [30–32]. At the same time, NSCs may generate neurotrophic factors that promote the recovery of impaired tissues in the damaged brain region [33–35]. As previously reported, MSCs utilized in the model of experimental stroke have elevated the functional recovery of neurological deficits resulted from cerebral ischemia [27]. Besides, another study suggested that MSC transplantation may improve the functional recovery of patients with stroke patients in the absence of adverse side effects [36]. Consistent with these findings, we found that BMSCs is helpful for the treatment of CP to some extent.

In addition, our study also demonstrated that BMSCs modified by XIAP improved the neurobehavioral situation, decreased content of Ach and increased activity of AchE, attenuated brain pathological injury, inhibited apoptosis of brain nerve cells and the activation of astrocytes in CP rats. Several studies have elucidated that in an animal model of cerebral hypoxic-ischemic injury, apoptosis is known as a delayed process of neuronal cell death [37, 38]. The upregulation of XIAP suppresses ischemic damage in the hippocampus and also recovers neurologic function of adult rat after global ischemia [39]. Wang et al. have stated that the activities of caspase-3 and -9 were virtually abolished; and relative to wild-type mice after hypoxicischemic injury, and XIAP neonatal transgenic mice exhibited decreased tissue loss [40]. In line with the results in our study, neuronal XIAP expression is increased in both developing brain of human and rat after ischemic injury [17]. Meanwhile, overexpression of XIAP in the penumbra of subacute infarcts has been suggested to play a role in the suppression of both caspase-dependent and -independent apoptosis in the position of ischemic developing brain [41–43]. Furthermore, it has been revealed that PI3K/Akt/XIAP axis is implicated in the apoptosis resistance of HL-60 cells in the condition of co-culturing with BMSCs through direct cell contact, and the inhibition of PI3K/Akt/XIAP axis may provide a promising therapeutic strategy for the treatment of acute myeloid leukemia [21]. All these aforementioned confirmed the important binding role of XIAP and BMSCs.

XIAP is a major member of the family of endogenous inhibitors of apoptosis (IAPs), which achieves anti-apoptosis by inhibiting different caspases in vitro and in vivo [44]. XIAP is the strongest caspase inhibitor in the IAP family [45]. Ischemic brain injury is a frequent and critical disease of the nervous system. Cerebral ischemia is often accompanied by a large number of neuronal apoptosis, which is closely related to the caspase family. Eberhardt et al. have found that XIAP gene was transfected into rat brain substantia nigra cells by adenovirus in an mitochondrial permeability transition pore-induced animal model of Parkinson’s disease. To some extent, up-regulation of XIAP can reduce the death of substantia nigra cells and protect dopamine neurons [46]. Simons and Wagenknecht have found that increased expression of XIAP in cerebellar granule cells and glial cells can reduce the damage of such nerve cells. It is speculated that XIAP may have strong anti-apoptotic effect in the central nervous system [47, 48]. BMSCs not only participate in the repair of neurological function of ischemic brain injury, but also serve as a carrier for gene therapy of cerebral ischemia, while whether overexpression of XIAP in BMSCs can prevent apoptosis in vitro remains to be unearthed, which could be verified in future study if condition permitting (Additional file 1).

## Conclusion

In summary, this present study demonstrates that BMSCs modified by XIAP can alleviate the development of CP caused by cerebral ischemia and hypoxia in rats. However, the mechanism of action underlying BMSCs therapy remains to be fully elucidated, and this persisting gap in our knowledge need further verification.

## Supplementary information

**Additional file 1.** The role of XIAP gene in brain damage of cerebral palsy rats and its molecular mechanism.

## Data Availability

Not applicable.

## Abbreviations

XIAP: X-linked inhibitor of apoptosis protein
BMSCs: bone marrow mesenchymal stem cells
CP: cerebral palsy
Ach: acetylcholine
AchE: activity of acetylcholinesterase
CP: cerebral palsy
SD: Sprague–Dawley
GFP: green fluorescent protein
RT-qPCR: reverse transcription quantitative polymerase chain reaction
Ad-GFP-XIAP: adenoviral vector carrying the XIAP gene
HE: hematoxylin-eosin
AI: apoptotic index
PFA: phosphofructaldolase
GFAP: glial fibrillary acidic protein
DAB: diaminobenzidine
cDNA: complementary DNA
AMV: avian myeloblastosis virus
GAPDH: glyceraldehyde phosphate dehydrogenase
BCA: bicinchoninic acid
PVDF: polyvinylidene fluoride
BSA: bovine serum albumin
ELISA: enzyme-linked immunosorbent assay
ANOVA: one-way analysis of variance
LSD-t: least significant difference t test
